# Tensile-shear properties of steel-Al adhesively bonded dissimilar joints and the effect of Al plate thickness

**DOI:** 10.1038/s41598-023-47072-1

**Published:** 2023-11-14

**Authors:** Yoshihiko Uematsu, Yoshihiro Ozeki, Paul Dario Toasa Caiza

**Affiliations:** 1 Department of Mechanical Engineering, Gifu University, 1-1 Yanagido, Gifu, 501-1193 Japan; 2 Graduate School of Natural Science and Technology, Gifu University, 1-1 Yanagido, Gifu, 501-1193 Japan; 3 Research Centre for Steel, Karlsruhe Institute of Technology, Otto-Ammann-Platz 7, 76131 Karlsruhe, Germany

**Keywords:** Mechanical engineering, Mechanical properties

## Abstract

Lap shear dissimilar joints between aluminium (Al) alloy, A6061-T6, and stainless steel, type 304, were fabricated by adhesive bonding. Three Al plates with different thicknesses were used to investigate the effect of the Al plate thickness on the tensile-shear properties, namely the effect of bending stiffness of Al plates. The maximum tensile-shear loads increased with increasing Al plate thicknesses. The fracture through the adhesive layer (cohesive fracture) occurred when the Al plate was the thickest, while the interface fracture between Al plate and adhesive layer appeared on the fracture surface with decreasing Al plate thickness. Fatigue strengths also increased with increasing Al plate thickness. When the fatigue strengths were normalized by the tensile strengths, the effect of the plate thickness became negligible. FEM analyses revealed that the stress concentration at the edge of adhesive on Al side decreased with increasing Al plate thickness, which could be related to the dependence of tensile and fatigue properties on the Al plate thickness.

## Introduction

In recent years, vehicle weight reduction has been promoted from the viewpoint of environmental problems and energy saving, and the thinning and weight reduction of automobile components are being promoted. Steel plates were mainly used for the automobile components, while conventional steels have been increasingly replaced by the other lightweight materials such as aluminium (Al) alloys and carbon fibre reinforced plastics (CFRP). Consequently, it is required to achieve a multi-material design concept that provides weight reduction and high functionality by using multiple materials together and selecting the right material for the right place^1–3^. Under such circumstances, the development of dissimilar materials joining methods should be developed, and the strength evaluation of the joints is attracting attention. Joining methods for dissimilar materials include conventional mechanical joining such as screws, bolts, and rivets^4–7^, metallurgical joining such as fusion welding^8–14^ and friction stir welding (FSW)^15–27^, which is a solid-phase joining method. However, adhesive bonding will be mostly widely used for the dissimilar materials joining^28–30^. That is because compared to the various joining methods mentioned above, adhesive bonding does not require complicated equipment used for FSW, and does not need a space for tightening screws, which can contribute to weight reduction, miniaturization, and labour saving. In addition, it can be applied to a wide range of dissimilar materials such as metals, plastics, composites, and ceramics, which cannot be joined by conventional fusion welding. Because of those advantages, adhesive bonding is expected to be used more widely in the future to achieve multi-material design components. Consequently, understanding of tensile-shear properties including fatigue of adhesively bonded joints is important^31,32^. However, basic knowledge about adhesive bonding between dissimilar materials is insufficient. Especially in the case of dissimilar materials, the modulus of elasticity differs greatly depending on the combination, and the bending stiffness also changes depending on the plate thickness. But the effect of such characteristics on the quasi static and fatigue tensile-shear properties of the joints is not clear.

For the present study, dissimilar materials adhesive joints of Al alloy A6061-T6 and austenitic stainless steel type 304 plates were fabricated using Al plates with three different thicknesses. Subsequently, static and fatigue tensile-shear tests were performed to investigate the effect of thickness on the tensile-shear properties and fracture modes. Also, a finite element method (FEM) was used to investigate the effect of Al plate thickness on the stress distribution in the adhesive layer.

## Experimental details

### Materials and specimens

**Figure 1:**
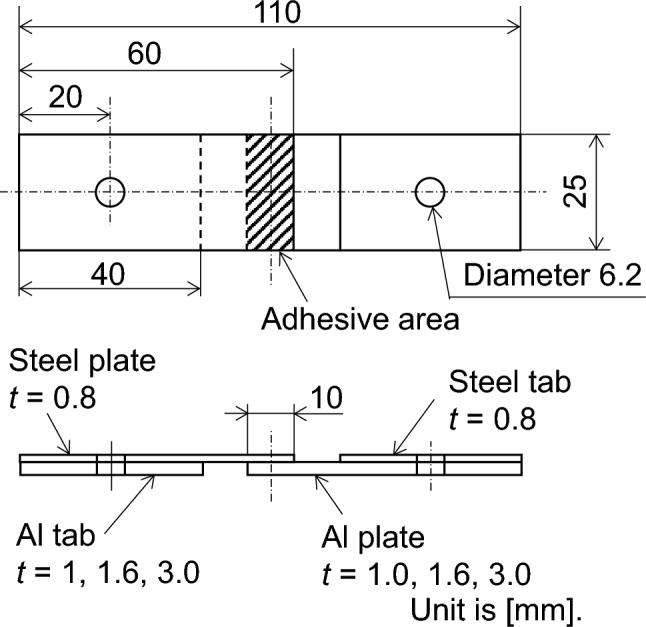
Tensile shear specimen configuration. The unit is in mm.

The test materials are austenitic stainless steel, type 304, and Al alloy, A6061-T6, plates. Figure 1 shows the lap-shear specimen configuration for the tensile and fatigue tests. As shown in the figure, the width and length of the specimen are 25 mm and 110 mm, respectively. The length of the overlap area, namely adhesive area, is 10 mm. The plate thickness of type 304 is fixed as 0.8 mm. On the other hand, Al plate thickness is set to be 1.0, 1.6, and 3.0 mm to investigate the effect of different bending rigidity. It should be noted that steel and Al tabs are bonded on Al and steel plates, respectively, which makes the thicknesses of the grip parts the same. When Al plate thickness is 1.0 mm, the bending stiffness is smaller than the steel plate, while 1.6 mm and 3.0 mm Al plates exhibit larger bending stiffness than the steel plate. Type 304 steel-steel similar joint is also fabricated for comparison. The adhesive for dissimilar joining is one-liquid thermosetting epoxy adhesive, SW-601, for automobiles supplied by Sunstar Engineering. The surfaces of the steel and Al plates were degreased by ethanol before bonding. The adhesive layer thickness is kept 150 µm. Glass beads with a diameter of 150 µm is mixed to the adhesive with the amount 1 wt% to keep the thickness constant. The heating temperature for curing the adhesive layer is 180 °C and holding time of 30 min. The edge of adhesive layer was cut by a thin razor to shape the adhesive fillet.

Shimadzu electro-hydraulic servo type fatigue testing machine with a capacity of 20 kN was used for the tensile and fatigue tests. Tensile tests were performed under the displacement control at a tensile speed of 5 mm/min. Fatigue tests were conducted in laboratory air at a frequency *f* = 10 Hz and a stress ratio of *R* = 0.1. The test condition is ambient temperature in laboratory air. To analyse the fracture morphology after the fatigue test, the fracture surfaces were observed using an optical microscope and a scanning electron microscope (SEM).

### FEM analyses

**Figure 2:**
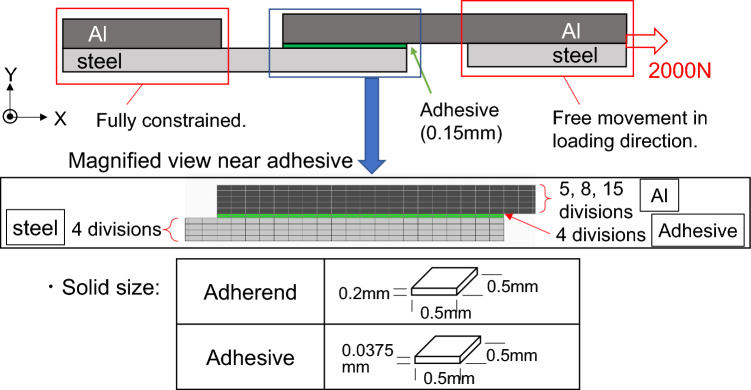
Schematic illustration of analytical model.

**Figure 3:**
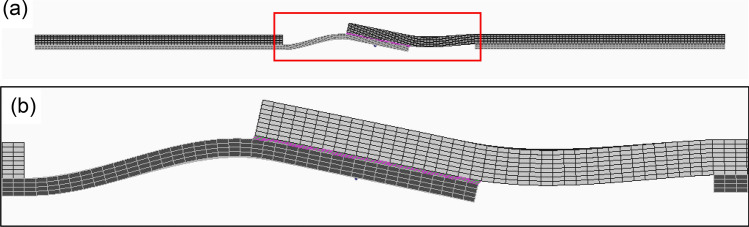
An example of deformed joint with the combination of Type304 and 1.6 mm thick Al plate; (**a**) General view, (**b**) Magnified view around the overlap area.

Stress distribution in the adhesive layer was calculated by a finite element method (FEM) using NX Nastran and Femap. As schematically shown in Fig. 2, one grip part is fully constrained, and the loaded grip side is constrained except for the direction of load application. Plastic deformation is not taken into account because the principal stress near the edge of the adhesive layer could be a controlling factor of fracture under tensile-shear condition. The effect of glass beads was neglected because the weight fraction of glass beads is small. The load of 2 kN was applied on the side surface of the grip part. The square solid size is 0.5 mm, and the solid thickness for the adherend was 0.2 mm, and that for the adhesive was 0.0375 mm. Therefore, the thicknesses of both steel plates and adhesive are divided into 4 layers. The basic concept of this analytical procedure is based on our previous study^33^. The division of Al plate thickness is 5, 8, and 15 depending on the thickness of Al plate. Table 1 shows the mechanical properties of the adherend and adhesive used in the analysis. As schematically shown in Fig. 3 (steel-Al_1.6_ joint. Subscript represents the thickness of Al plate.), the rotation around the overlap area is taken into account. It should be noted that the deformation was enlarged 100% in Fig. 3 to clarify the rotation deformation.

**Table 1:** Mechanical properties.

Material	Young’s modulus (MPa)	Poisson’s ratio
Type 304	194,000	0.30
A6061-T6	72,300	0.30
Adhesive	4500	0.36

## Results and discussion

### Tensile properties

**Figure 4:**
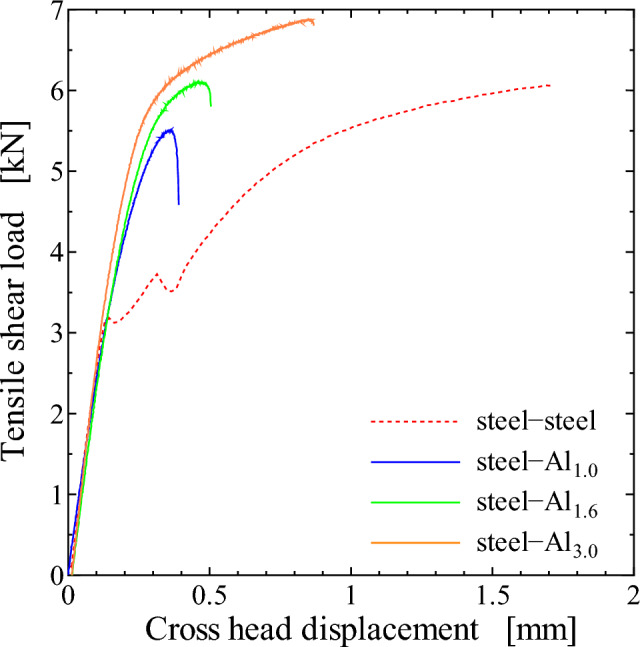
Relationship between tensile-shear load and cross head displacement.

**Figure 5:**
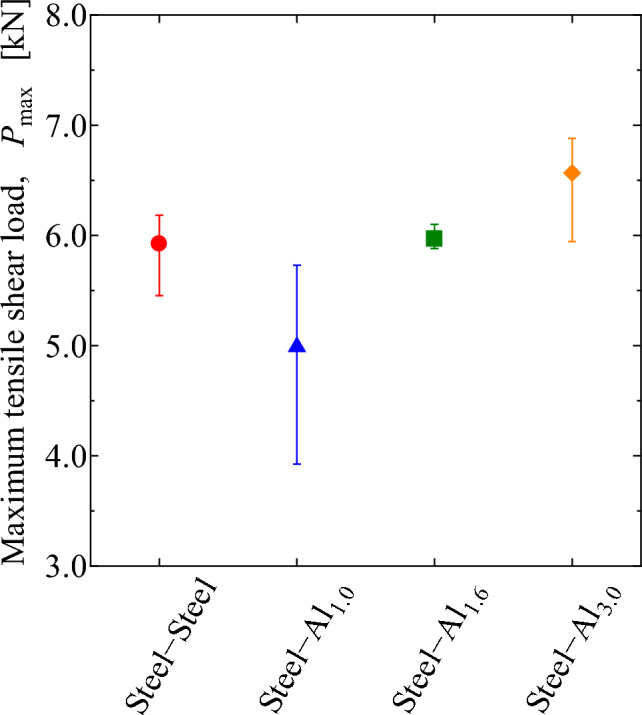
Maximum tensile-shear loads of similar and dissimilar joints.

Figure 4 shows the typical relationships between tensile-shear load and cross head displacement. Cross head displacement contains slipping at the grip parts, while it can be said that the elastic response is kept until the load reaches 3 kN. Figure 5 summarizes the tensile-shear strength of each specimen, which is the average of three samples. The result of steel-steel similar joint is also shown in the figure. The maximum tensile-shear load of steel-steel similar joint is nearly the same with that of the steel-Al_1.6_ dissimilar joint. In the case of the steel-Al dissimilar joints, the tensile strength increased as the thickness of Al plate increased. It should be noted that the loading axes of the joint with different plate thicknesses does not align with the mid-planes of the adhesive layers, and the misalignment increases with increasing Al plate thickness in the present case. The misalignment will have detrimental effect on the tensile properties because of the secondary bending moment induced by the misalignment. In the present case, however, tensile strength increased with increasing Al plate thickness despite the larger bending moment. It implies that the effect of secondary bending moment due to the misalignment might be smaller than the effect of the rotation shown in Fig. 3.

**Figure 6:**
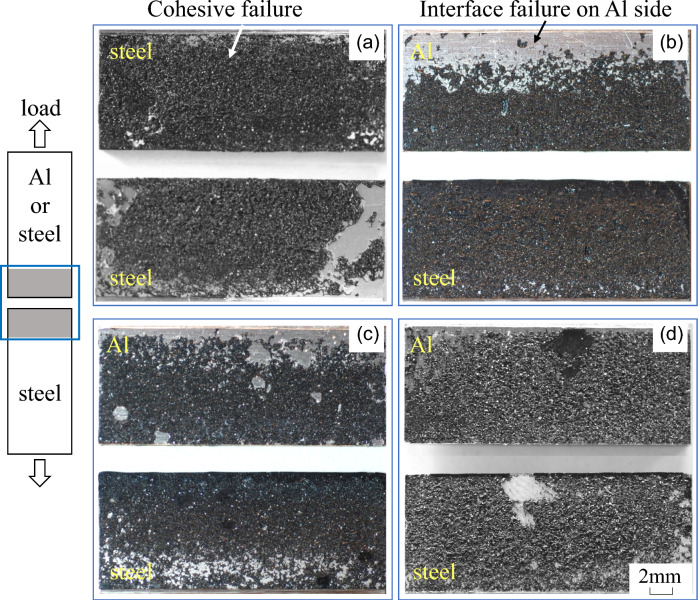
Fracture surfaces of tensile shear tests: (**a**) stee-steel, (**b**) steel-Al_1.0_, (**c**) steel-Al_1.6_, (**d**) steel-Al_3.0_.

**Figure 7:**
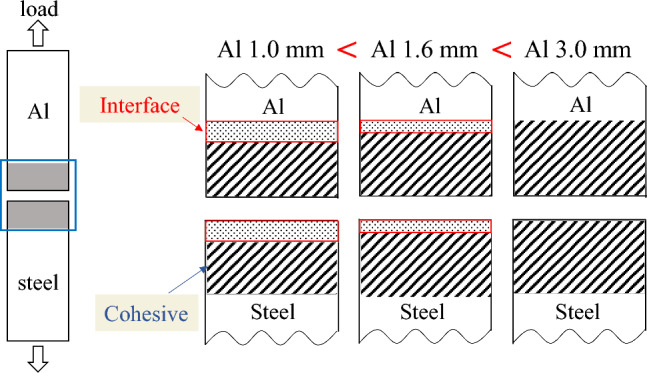
Schematic illustration showing the dependence of interface failure on Al plate thickness.

Figure 6 shows fracture surfaces of the tensile-shear test specimens. The cohesive fracture, which is the fracture of the adhesive, was dominant in steel-steel similar joint (Fig. 6a), where both upper and lower fracture surfaces are mainly covered by the failed adhesive. In the case of dissimilar joints, interfacial fracture was observed at the edge of the adhesive layer on the Al side in steel-Al_1.0_ specimen as shown by the arrow in Fig. 6b. It reveals that the interface fracture occurred at the interface between Al and adhesive, and not at the steel side interface. The interfacial fracture was also observed on Al side interface of steel-Al_1.6_ dissimilar joint. However, as schematically shown in Fig. 7, it should be noted that the interfacial fracture area became smaller with increasing Al plate thickness. Consequently, cohesive fracture is dominant in steel-Al_3.0_ joints. However, interfacial fracture was clearly observed on the Al-side interface near the edge of the bonded area in steel-Al_1.0_ and steel-Al_1.6_ joints. Therefore, it is assumed that the tensile fracture had started at the edge of the bonded area, and the stress near the Al side interface in adhesive might be the controlling factor of the tensile strength and fracture mode (cohesive or interface fracture). Subsequently, stress distribution in the adhesive is analysed in the next section.

### Stress analysis of adhesive

**Figure 8:**
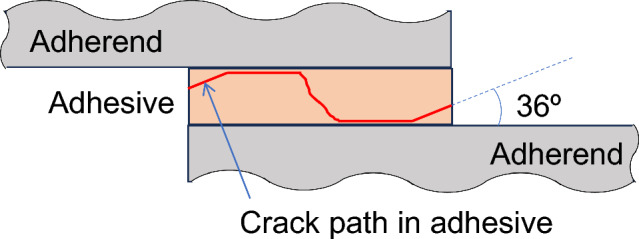
Schematic illustration showing crack path in adhesive^34^.

**Figure 9:**
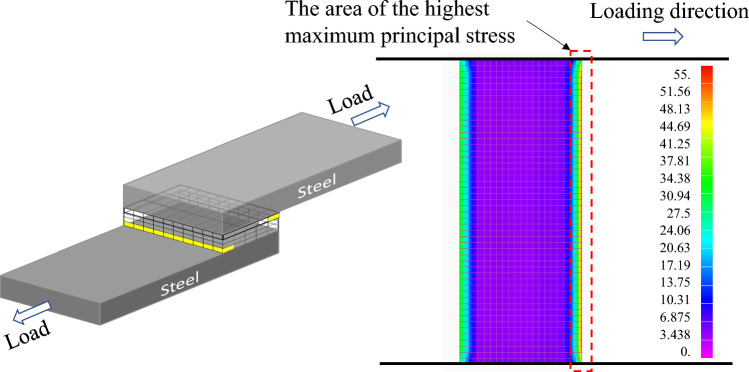
Maximum stress distribution in the adhesive layer in steel-steel similar joint.

**Figure 10:**
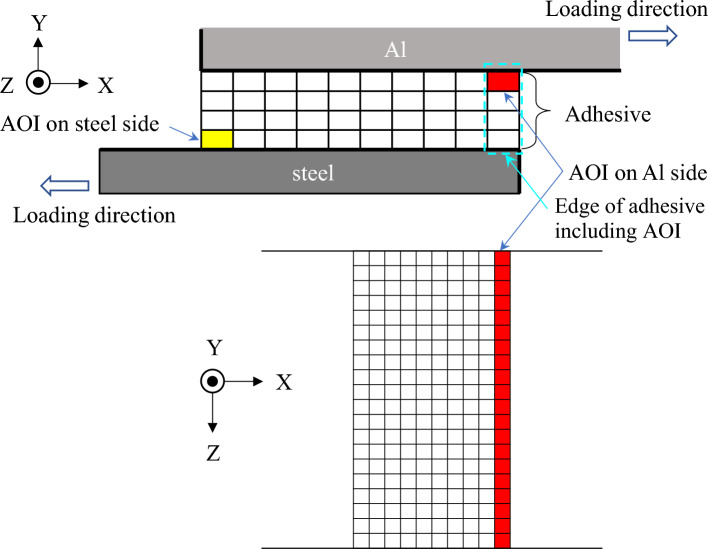
Area of interest (AOI) on Al side and steel side where maximum principal stress is analyzed.

As typically shown in Fig. 3, bending moment occurs in the adherends in single-lap joints. In our previous study, the geometry of the edge of adhesive was investigated on the fatigue fracture surface in detail using laser scanning microscope^34^. It revealed that the angle of the adhesive at the start of fatigue failure and early stage of fatigue crack growth was mainly related to the principal stress as schematically shown in Fig. 8. Therefore, the principal stress at the edge of adhesive, in which bending of adherend was taken into account, was used for the discussion as described in the “FEM analyses” section. Figure 9 shows the FEM analytical result of steel-steel similar joint. Right-hand counter diagram indicates the maximum principal stress distribution in the layer just below the upper steel plate. It should be noted that the maximum principal stress was the largest at the edge of the adhesive layer on the loading side, which is shown by the yellow cells in the left-hand schematics. Figure 10 indicates the areas of interest (AOI). The maximum principal stresses at the edges shown by red solids on Al side and yellow ones on steel sides were used for the evaluation, namely the edges at the upper and lower loading sides.

**Figure 11:**
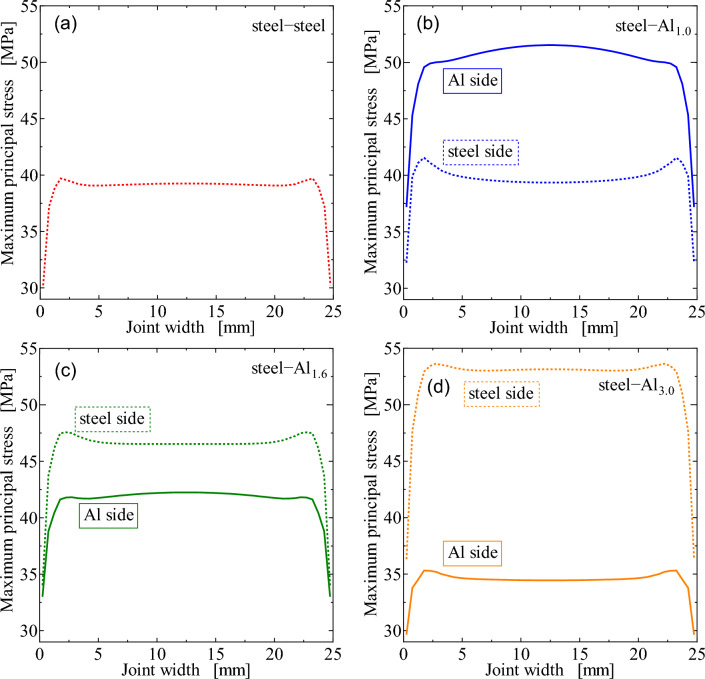
Maximum principal stress distribution as a function of joint width: (**a**) steel-steel, (**b**) steel-Al_1.0_, (**c**) steel-Al_1.6_, (**d**) steel-Al_3.0_.

**Figure 12:**
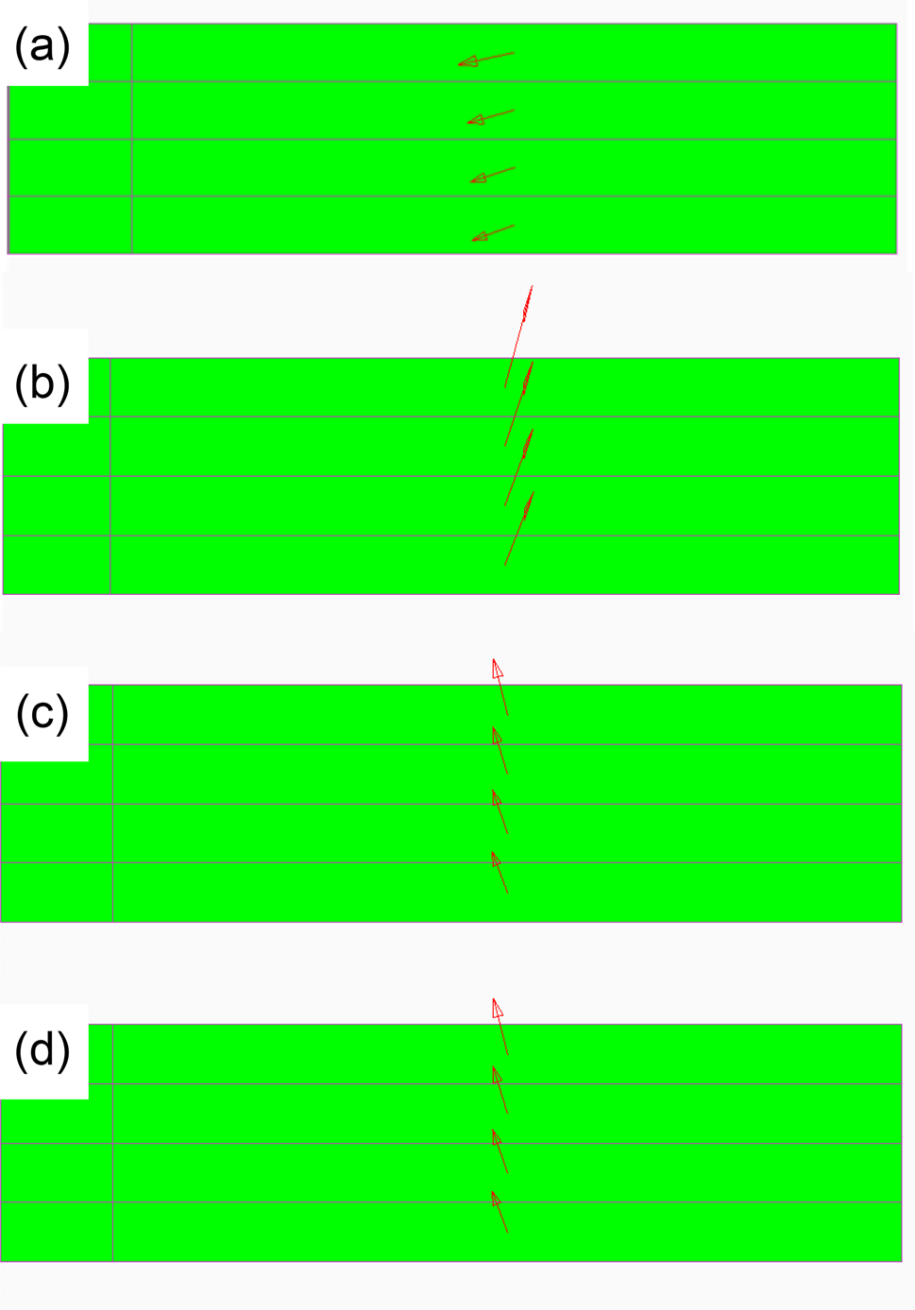
Direction of maximum principal stress at the edge of adhesive including the AOI on Al side shown in Fig. 10: (**a**) steel-steel, (**b**) steel-Al_1.0_, (**c**) steel-Al_1.6_, (**d**) steel-Al_3.0_.

Figure 11 shows the maximum principal stress distributions in the AOI of steel-Al dissimilar joints with the different thicknesses of Al plates. The result of steel-steel similar joint is also shown as Fig. 11a. As mentioned above, the stresses at the edges on Al and steel loading sides are plotted as a function of joint width. The solid and dotted lines represent the stresses at the edges on Al and steel sides, respectively. In steel-Al_1.0_ dissimilar joint, the stress is much higher on Al side. However, with increasing the thickness of Al plates, the stress on steel side increases, and that on Al side decreases. The stress on steel side becomes much higher when the thickness of Al plate is 3.0 mm. These results imply that the fracture locations can be dependent on the thicknesses of Al plates. Figure 12 shows the direction of maximum principal stress at the edge of adhesive including the AOI on Al side shown in Fig. 10. It should be noted that the maximum principal stress is nearly shear direction in steel-steel similar joint while it becomes perpendicular to the interface in steel-Al dissimilar joints. This transition of direction is one of the reasons why interface failure occurred in the dissimilar joints (Fig. 7).

In the case of steel-Al_1.0_ and steel-Al_1.6_ joints, interface fracture occurred on Al side as schematically shown in Fig. 7, which implies that the fracture had started at the edge of adhesive on Al side. Seeing Fig. 11, the stress in the adhesive on Al side decreases with increasing the thickness of Al plate, which correlates well with the dependence of the maximum tensile-shear load on the thickness of Al plate (Fig. 5). It should be noted that the stress in steel-steel similar joint shown in Fig. 11a is nearly comparable with that on Al side in steel-Al_1.6_ joint in Fig. 11c, which corresponds to the fact that the tensile-shear loads are nearly comparable between steel-steel similar and steel-Al_1.6_ dissimilar joints. It should be noted that the FEM analyses are elastic, where applied load is 2 kN. As shown in Fig. 4 of the load-cross head displacement curves, actual fracture occurred at the loads much higher than elastic limit. However, it is considered that the elastic FEM analytical results are strongly related to the actual dependence of maximum tensile-shear loads on the thickness of Al plate. Consequently, the highest tensile-shear load of steel-Al_3.0_ could be attributed to the suppression of rotation around overlap area (Fig. 3), because the cohesive fracture was dominant in this joint (Fig. 6d).

### Fatigue strength

**Figure 13:**
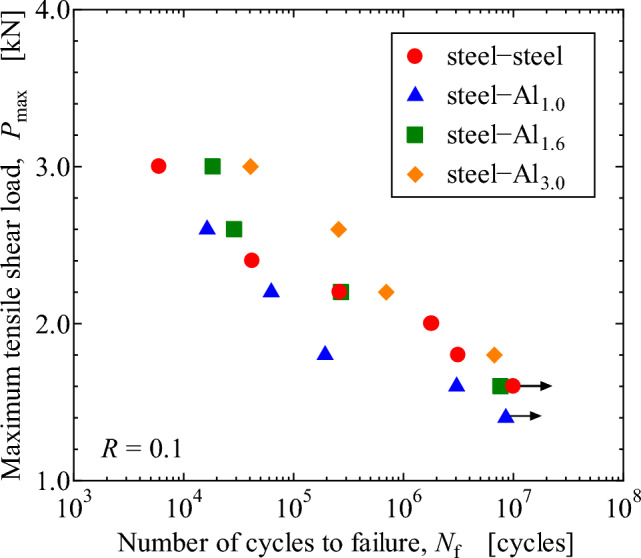
Relationship between maximum tensile shear load, *P*_max_, and number of cycles to failure, *N*_f_.

**Figure 14:**
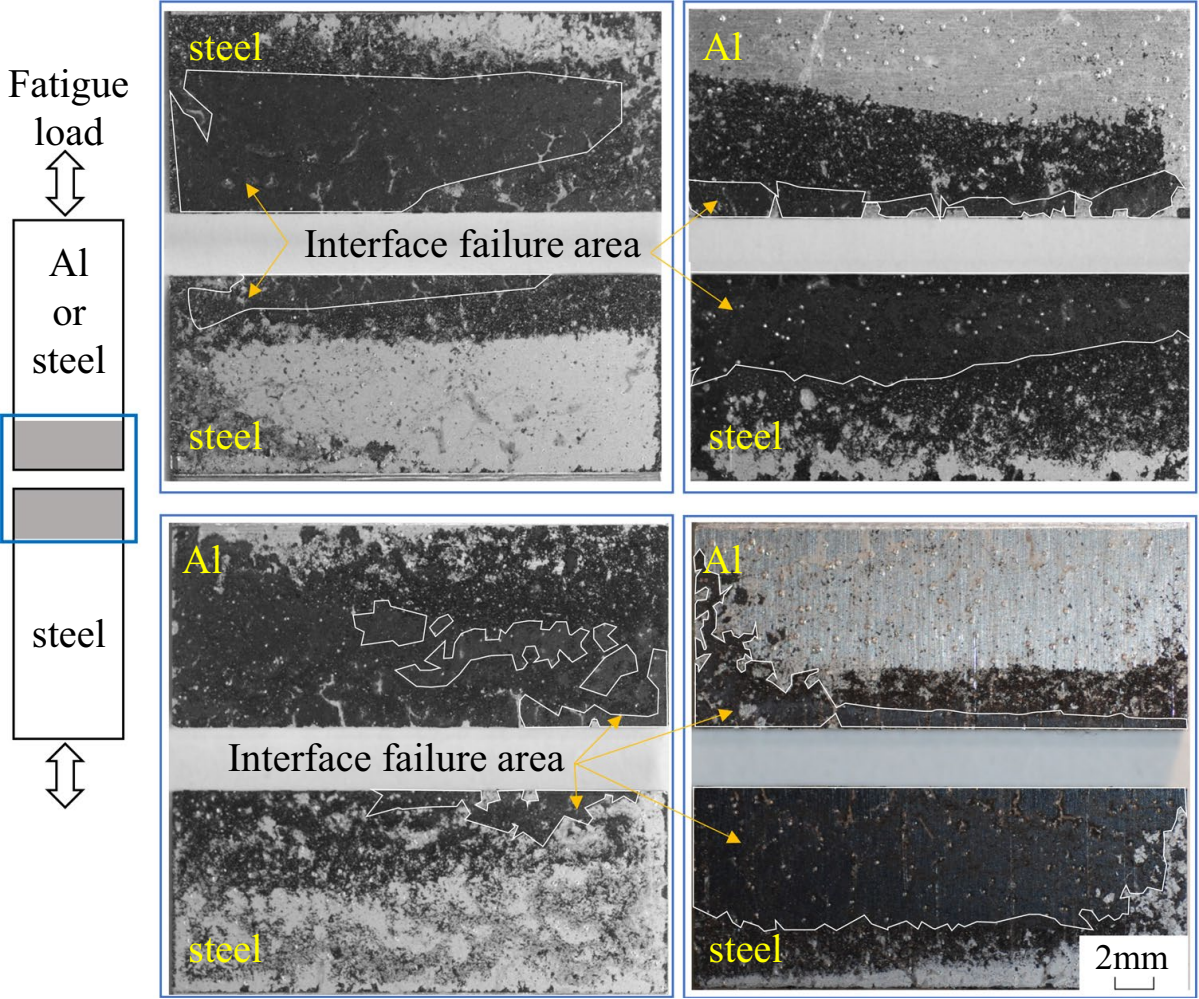
Fracture surfaces of tensile shear fatigue tests at the maximum load, *P*_max_, of 2.2 kN: (**a**) stee-steel (*N*_f_ = 2.6 × 10^5^), (**b**) steel-Al_1.0_ (*N*_f_ = 6.2 × 10^4^), (**c**) steel-Al_1.6_ (*N*_f_ = 2.7 × 10^5^), (**d**) steel-Al_3.0_ (*N*_f_ = 6.9 × 10^5^).

Figure 13 shows the relationship between tensile-shear maximum load, *P*_max_, in fatigue loading and number of cycles to failure, *N*_f_. Similar to the static tensile-shear strength, the fatigue strength increases with increasing the thickness of Al plate. Fatigue fracture surfaces at *P*_max_ = 2.2 kN are revealed in Fig. 14. It should be noted that interface fracture, which is shown as the enclosed areas by the white solid lines, is clearly seen in all joints, even in steel-steel similar and steel-Al_3.0_ dissimilar joints, in which cohesive fracture was dominant under quasi static loading condition. It indicates that fatigue crack initiated at the edge of adhesive and predominantly propagated along the interface of adhesive. As shown in Fig. 14, interface failure between adhesive and Al plate occurred during fatigue crack growth, thus peeling strength or shear strength seem important. But as mentioned in the “Stress analysis of adhesive” section and Fig. 8, the fatigue crack initiation and early growth are related to the principal stress^34^, which indicates the analyses of principal stress is more important. It should be noted that the upper limit of *P*_max_ in fatigue test is 3 kN, which is the elastic limit in Fig. 4. Therefore, elastic FEM analyses can be reasonably related to the fatigue test results.

**Figure 15:**
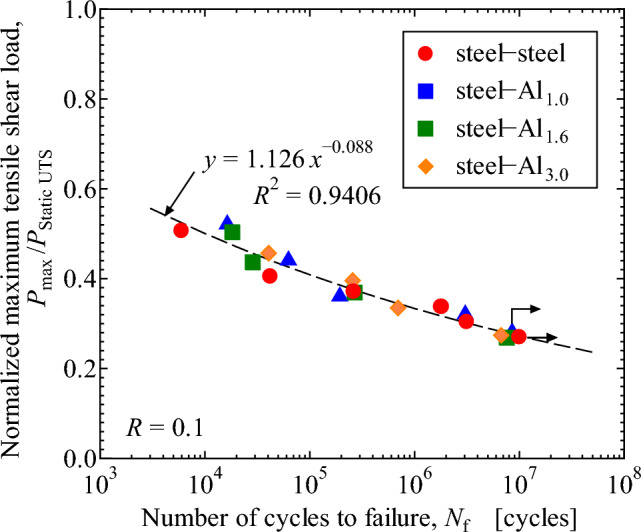
Relationship between normalized maximum tensile shear load, *P*_max_/*P*_static UTS_, and number of cycles to failure, *N*_f_.

*P*_max_ in the fatigue test is normalized by the tensile-shear load in the quasi static test, and the results are revealed in Fig. 15. The effect of the thickness of Al plate does not play an important role in Fig. 15, which indicates that the approximate curve shown by the broken line in the figure with the coefficient of determination, *R* of 0.941 can be used as a master design fatigue curve of dissimilar joints between steel plate with the thickness of 0.8 mm and Al plates with different thicknesses. In the fatigue tests, load levels are in the elastic range in Fig. 4, thus the effect of bending might be small. In the quasi-static tests, bending moment becomes severer in the un-elastic range in Fig. 4. However, the normalization of the fatigue results by tensile loads still gives good correlation as shown in Fig. 15, indicating that the bending moment effect can be neglected in such a normalized curve. Consequently, quasi static tensile test and fatigue tests using steel-Al dissimilar joint with one Al plate thickness can predict fatigue strengths of steel-Al dissimilar joints with different Al plate thicknesses. Furthermore, a simple elastic FEM analysis can qualitatively estimate the dependence of tensile-shear strengths on the thickness of Al plate. Those findings are important from the viewpoint of fatigue design of steel-Al plates dissimilar adhesive joints.

## Conclusions

Type 304 stainless steel plates with the thickness of 0.8 mm were adhesively bonded to Al alloy, A6061-T6, plates with different thicknesses of 1.0 mm, 1.6 mm and 3.0 mm. Quasi static and fatigue tensile-shear tests were performed to investigate the effect of Al plate thickness on the tensile-shear loads and fatigue strengths. Stress analyses by FEM were performed to investigate the correlation between stress distribution in the adhesive and tensile-shear properties. The main results obtained are as follows.

1. The tensile-shear loads under quasi static loading conditions increased while Al sheet thickness increased. Fractographic analyses revealed that interface fracture occurred in steel-Al_1.0_ and steel-Al_1.6_ joints, where the interface fracture area on the fracture surfaces decreased, and the Al plate thickness increased. Cohesive fracture was dominant in steel-steel similar and steel-Al_3.0_ dissimilar joints.
2. FEM analyses revealed that the highest maximum principal stress appeared at the edge of adhesive layer on the loading side. The edges of adhesive layer on the loading side were defined as AOI, and the dependence of maximum principal stress on the thickness of Al plate was clearly recognized. The maximum principal stress on Al side decreased while the thickness of Al plate increased, and it was correlated well with the actual dependence of the tensile-shear load on Al plate thickness.
3. The fatigue strength increased while the thickness of Al plates increased similar to quasi static tensile-shear strength. When the maximum tensile-shear load, *P*_max_, in fatigue test was normalized by the quasi static tensile-shear strength, one master curve was obtained. That curve could be used as a fatigue design curve of steel-Al dissimilar adhesive joints with different Al plate thicknesses.

## Data Availability

All data generated or analyzed during this study are included in this published article.
